# Oral Complications and Management Strategies for Patients Undergoing Cancer Therapy

**DOI:** 10.1155/2014/581795

**Published:** 2014-01-08

**Authors:** Hai Ming Wong

**Affiliations:** ^1^Faculty of Dentistry, The University of Hong Kong, Hong Kong; ^2^The Prince Philip Dental Hospital, 34 Hospital Road, Hong Kong

## Abstract

With cancer survival rate climbing up over the past three decades, quality of life for cancer patients has become an issue of major concern. Oral health plays an important part in one's overall quality of life. However, oral health status can be severely hampered by side effects of cancer therapies including surgery, chemotherapy, radiotherapy, and hematopoietic stem cell transplantation. Moreover, prevention and treatment of these complications are often overlooked in clinical practice. The present paper aims at drawing health care professionals' attention to oral complications associated with cancer therapy by giving a comprehensive review. Brief comments on contemporary cancer therapies will be given first, followed by detailed description of oral complications associated with cancer therapy. Finally, a summary of preventive strategies and treatment options for common oral complications including oral mucositis, oral infections, xerostomia, and dysgeusia will be given.

## 1. Introduction

Cancer, from its initial diagnosis to the completion of its treatment, is a heart-rending experience for many. It is a period of great pressure and stress for not only the patients themselves but also their families and friends. According to the United States Cancer Statistics 2009, the National Program of Cancer Registries, the ten ranking cancers by site in the American population (per 100,000 persons) are prostate (137.7), breast (123.1), lung and bronchus (64.3), colon and rectum (42.5), corpus and uterus, NOS (25.1), urinary bladder (20.5), melanomas of the skin (19.4), non-Hodgkin's lymphoma (18.9), kidney and renal pelvis (15.7), and thyroid (13.2) [1]. The American Cancer Society has estimated that there will be approximately 1,660,290 new cancer cases being diagnosed in 2013. As the second leading cause of death in the United States, preceded only by heart disease, cancer is expected to take the lives of 580,350 Americans in the year of 2013, that is, about 1600 deaths per day [2, 3]. Though the survival statistics can vary significantly with the stage at which the cancer is diagnosed as well as the cancer type itself, survival rates continued to climb over the past years. In fact, the 5-year relative survival rate for all cancers diagnosed from 2002 to 2008 is 68%, compared to 49% in 1975–1977 [3]. Coupled with the ongoing advances in cancer detection and treatment modalities, the future trend would entail increased likelihood for dentists to encounter patients who are currently, or have previously been, under cancer therapy.

Contemporary cancer treatment modalities commonly include surgical resection, chemotherapy, radiotherapy, and hematopoietic stem cell transplantation (HSCT), a form of immunotherapy, either administered alone or used in combination. Although the effectiveness of cancer treatment has continued to improve over the past decades, collateral damage to the head and neck structures is frequently encountered as an unwanted consequence. Radio- and chemotherapy can cause direct harm to the soft and hard tissue of the oral structures, whereas their systemic toxicity can give rise to indirect damages. These oral complications, be they acute or chronic, may arise throughout and after cancer treatment and often encompass mucositis, dysgeusia, and infectious diseases [4]. Although literature has shown that the oral health status in most cancer patients mirrors those of the general population, poorly restored dentition, moderate to advanced periodontal disease, and other pathologies associated with negligence of oral health care, the many oral complications as sequelae of aggressive cancer treatment can greatly hamper patients' quality of life; this necessitates optimal oral health care [5]. Maintaining oral health is essential in the preservation of daily functions, such as eating, verbal and nonverbal communications, and the prevention of infectious diseases.

While the necessity of dental clearance is debatable and empirical guidelines vary from center to center, assessment, treatment, and prevention of any preexisting pathological condition make up an important aspect of the overall treatment outcome in cancer patients [6, 7]. Unfortunately, priority is often given to the more “life-threatening” condition that is cancer, and administering oral care has become an activity frequently neglected [8, 9]. Yet, it is the ethical and medical/legal responsibility of all health care practitioners, including professionals in the field of dentistry, to ensure that the oral health status of patients undergoing cancer therapy is thoroughly evaluated. Thus, it is the aim of this paper to paint a detailed picture for dental professionals on the topic of oncology related oral care. Cancer treatment modalities will be introduced first and oral complications of cancer therapy will be discussed. Some therapeutic alternatives that may help to alleviate the painful and debilitating adverse effects will also be presented.

## 2. Management of Malignancy

Treatment of malignancy is complex and often involves a multidisciplinary approach. It is beyond the scope of this paper to give an in-depth review of different types of cancer therapy. Brief comments on cancer treatment modalities, including surgery, chemotherapy, radiotherapy, and HSCT, will be made to give a general picture of contemporary mainstream cancer therapies.

Surgery has come a long way in the treatment of malignancy and is still one of the most common methods in managing primary solid tumors today [10]. In dentistry, the impacts of surgical resection on patients' lives are particularly evident in the cases of oral and oropharyngeal cancers. Over the past decades, advances in surgery have resulted in major changes in surgical approaches to the mandibular areas and cervical lymph nodes [11]. In addition, safer anesthesia, tumor ablation via radiofrequency, and radiosurgery have opened up many more options and alternatives for the professionals in the effective management of the disease [12]. Combined with advances in various reconstruction techniques, the functional and aesthetic outcomes have been greatly improved in cancer patients. Nowadays, survivors of head and neck cancers no longer have to live with severe functional disabilities or aesthetic impairments that compromise their quality of life [11].

Chemotherapeutic agents are drugs designed to have a selective toxicity towards the tumor cells. Current chemotherapeutic agents are often cytostatic or cytotoxic in nature to prevent the rapid division of the malignant cells and/or destroy them in the process [13]. Unlike surgery and radiation therapy, whose usages are limited to cancers confined to specific areas of the body, the major advantage offered by chemotherapy is the ability to target widespread or metastatic cancer [14]. Yet, even the most modern chemotherapeutic agents are not without their shortcomings. Ironically, while exploiting the tumor cells' characteristic feature of shortened cell cycle and having a selective toxicity design in mind, these anticancer agents are not so “specific” as they also act upon normal cells with a high turnover rate (accelerated cell cycle) such as bone marrow cells, hair follicle cells, and the epithelial cells of the gastrointestinal tract [13, 15]. Some common drugs used in chemotherapy for oral cancer include 5-fluorouracil, bleomycin, cisplatin, cyclophosphamide, methotrexate, and vinblastine [16].

As one of the most effective forms of cancer treatment, radiation therapy plays an indispensable role in the management of many head and neck cancers as well as lymphomas. The dose of ionizing radiation administered often depends on factors such as the location of the malignancy, the type of the malignancy, the sensitivity of the surrounding normal tissues, and whether radiation is used as the sole treatment option [17, 18]. Typically, for most head and neck cancer patients, a dose of 2 Gy per fraction is delivered once a day, five days per week, over a five-to-seven-week period with a total dosage of 64–70 Gy [19]. The amount of radiation used to treat malignant lymphomas is usually lower [17]. Although the main aim of radiotherapy is to deliver a concentrated and lethal dose of radiation to the solid tumor while at the same time minimizing the exposure of the surrounding tissues, the salivary glands, oral mucosa, and jaws are inevitably covered in the blast radius, resulting in a variety of problems within the area [20].

HSCT, also known as bone marrow transplant, has formed an important part of the treatment modalities for many malignant diseases, acute and chronic leukemia, myelodysplastic syndromes, myeloproliferative disorders, multiple myeloma, and non-Hodgkin's lymphomas, as well as a number of other nonmalignant diseases such as aplastic anemia [21, 22]. For patients to undergo the HSCT procedure, they are required to partake in preoperative conditioning which often involves the use of cyclophosphamide and total body irradiation (TBI). The objective is to eradicate the cancer and induce an immunosuppressive environment that permits engraftments. Although HSCT can result in more cures and remissions compared to alternative treatment, it also tends to cause greater morbidity and mortality with the mortality rates of less than 2% and 10% for some autologous and allogeneic transplantations, respectively. With as much as 40% of the advanced cancer patients dying from complications related to transplantation, further research is warranted to determine the best ablative regimens for specific conditions and reduce the toxicity of the preparative regimens [21].

## 3. Oral Complications of Cancer Therapy

### 3.1. General Considerations and Overview

Despite the encouraging evolvement in cancer management over the past decades, one should bear in mind that current treatment modalities do have the potential to result in debilitating and sometimes life-threatening adverse effects that not only decrease the patients' quality of life but also increase their morbidity and mortality.

Oral complications associated with cancer therapy encompass diseases such as stomatitis, infection, bleeding, mucositis, pain, loss of function, and xerostomia [23]. The mucosa, the periodontium, and the teeth are the three anatomical sites most commonly associated with these complications [24]. Oral complications associated with specific oncological therapy will be discussed in the following sections.

### 3.2. Complications Associated with Surgery

The long-term complications associated with the surgical treatment of oral cancers are many; they range from functional limitations on speech, mastication and swallowing, damages to the cranial nerves and the resultant neurological problems, chronic fistulas, and healing issues to aesthetic considerations such as severe disfigurement and prosthetic rehabilitation; taking these functional and aesthetic impairments, together with their psychological implications, the patients' long-term quality of life could be hampered [25].

**Figure 1:**
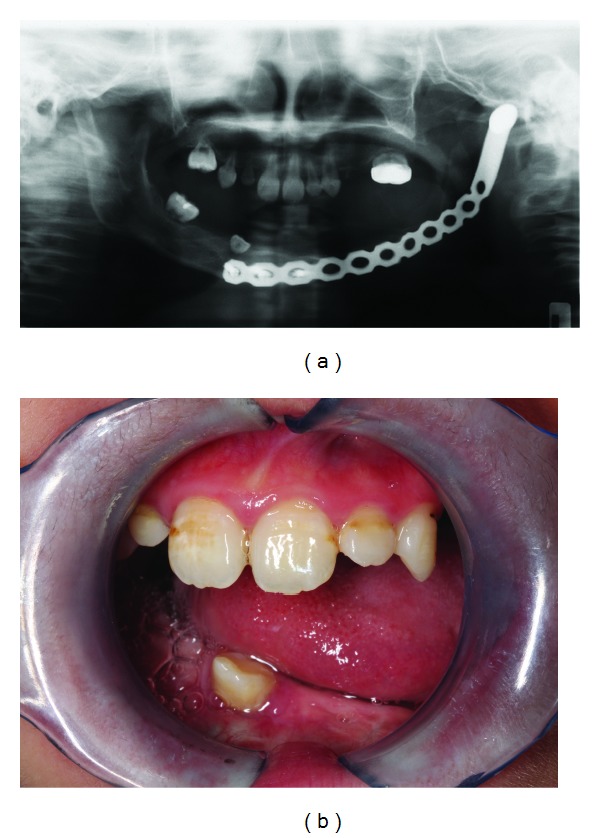
(a) Surgical resection involving part of the mandible in a patient with rhabdomyosarcoma. (b) Limited mouth opening after surgical resection.

Surgical resection of cancers in the oral cavity can negatively impact speech, mastication, and swallowing in a very significant way. In general, surgical ablation that involves the most anterior region of the tongue is often associated with significant speech alteration, whereas ablation that incorporates the posterior tongue affects swallowing [26–29]. With tumor ablation and the subsequent loss of a significant portion of the tongue, the manipulation and formation of the bolus (the oral preparatory phase) and the transfer of bolus from the anterior oral cavity to the posterior tonsillar area (the initiation of the swallowing reflex) are severely restricted [30]. The situation could be further complicated where surgical management also involves the floor of mouth, maxilla, and mandible with the adjacent tissues (Figure 1(a)); those are vital structures for mastication. Resection of the maxilla or the mandible could prove problematic for the patient during grinding as the stable and reproducible stomatognathic system relationships or tooth-to-tooth contact is lost, resulting in diminished biting force [30, 31]. Furthermore, combined with soft tissue bulk and sensation loss, the ability of the patient to manipulate food bolus to and fro from occlusal table and its consolidation before deglutition are impaired. Thus, the overall masticatory efficiency, which encompasses manipulation, trituration, and consolidation, being the result of synchronous interaction of both hard and soft tissues, is drastically reduced [32].

Trismus, that is, limited mouth opening, is a common complaint after oral cancer surgery (Figure 1(b)). Postoperative healing, including fibrosis and scar contraction, often results in restricted interocclusal opening of less than 35 mm between the maxillary and mandibular incisors. Procedures that may lead to trismus commonly include maxillary surgery involving the origin of medial and lateral pterygoid muscles from the pterygoid plates and mandibulectomy involving any of the muscles of mastication (the temporalis insertion to the coronoid process, the masseter insertion to the angle and ramus, and the pterygoid insertions to the medial ramus and condylar neck) [30]. Note that trismus could be exacerbated by the fibrotic changes due to combination radiotherapy.

Additionally, the resection of primary tumor and lymph nodes has put several cranial nerves at risk: spinal accessory nerve, phrenic nerve, hypoglossal nerve, lingual nerve, vagus nerve, sympathetic trunk, and marginal mandibular branch of the facial nerve. The size of the tumor and its location and the extent of the neck pathology often require the nerves involved or in close association to be sacrificed. The issue with access and certainty of satisfactory tumor removal can compromise the integrity of the cranial nerves within the area [30].

Fistula is another complication often associated with oral oncologic surgeries. The risk of fistulas being developed often depends on the general physical and nutritional status of the patient, the incision design, and the tumor type and stage. Its management is particularly difficult where radiation therapy is also involved, as surgical wound closure is delayed due to low oxygen tensions, vasculitis, endothelial fibrosis, and reduced blood supply [25]. Fistulas generally occur 3-4 weeks after surgery, but they can also develop as early as 1 week. Persistent or chronic fistulas are those that remain present 1 month after the surgery. In addition, patients may present with low grade fever, inflammation, and induration of the skin flap under the area of dependant drainage. Prevention is often the best treatment, but surgical excision and closure of the mucosa and skin are indicated where the problem persists [25, 33].

The complex anatomy of the bone and associated muscle attachments often require careful planning and placement of plates and screws to stabilize bone segments and secure bone flaps in mandibular osteotomies and/or resection. Any abusing of the reconstruction principles and overmanipulation of the material, along with unbalanced masticatory force, can result in hardware failure ranging from fracturing of the plates, loosening of the screws, and mobility of the mandibular segments to exposure of the material through the overlying soft tissue and secondary infection [25]. Although early treatment of the problem is preferred, surgical intervention may be delayed due to adjunctive radiation therapy which can compromise the wound healing; in which case, hyperbaric oxygen treatments may improve the healing abilities of the soft tissue overlying the hardware replacement [33–35].

Appropriate prosthetic and functional rehabilitation, which entails the reestablishment of a functional maxillomandibular complex providing for an adequate dentition for mastication with underlying bone support for facial features and soft tissue for the restoration of speech and swallowing, served as the desired endpoint for many patients. However, such a feat may not always be feasible as many factors play into this prosthetic problem. As noted by Kolokythas, the issue is a multidisciplinary one; it may not always be possible to convene all members of the treatment team to discuss the treatment plan of the oral cancer patient prior to resection. Thus, the plans for reconstruction may often have to be formed postoperatively and may not be ideal. Furthermore, the extent of the resection, several postsurgical and radiation associated complications, may not allow for the ideal rehabilitation. The patient's compliance and financial background may also be a barrier to reaching the final restorative goal [25].

### 3.3. Complications Associated with Chemotherapy

Chemotherapeutic agents have gained a notorious reputation in damaging not only the malignant cells but also the normal tissue in the patient's body. The level and the type of toxicity of the treatment greatly depend on the overall immune status of the patient prior to and during chemotherapy, the regimen itself, the frequency and the dosage of the treatment, the route of administration, and the type of tumor. In many patients, these drugs can cause a number of oral complications including mucositis, pain, infection, hemorrhage, xerostomia, and neurologic and nutritional problems [14].

**Figure 2:**
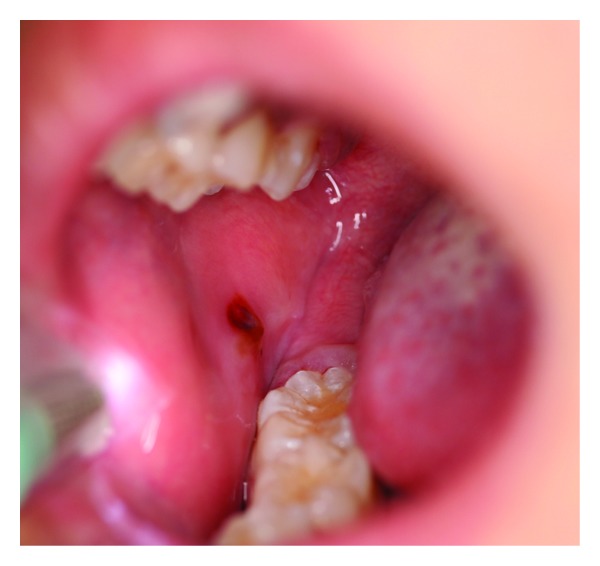
Localized buccal mucositis in a patient with osteosarcoma.

**Figure 3:**
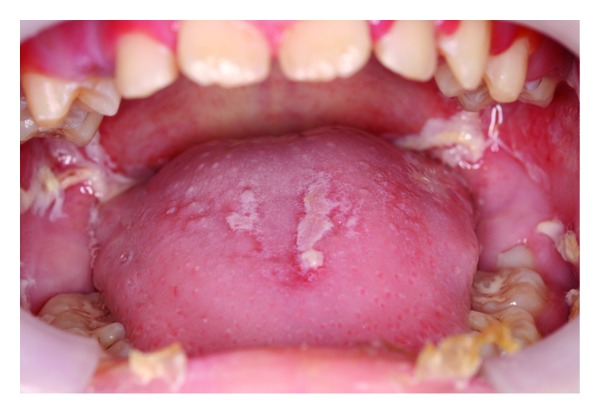
Generalized mucositis in a patient with acute myeloid leukemia.

Oral mucositis (OM) is an iatrogenic condition of erythematous inflammatory changes which tends to occur on buccal and labial surfaces, the ventral surface of the tongue, the floor of the mouth, and the soft palate of patients receiving chemotherapy [36]. Its severity ranges from localized (Figure 2) to generalized erythema (Figure 3) to frank ulceration and hemorrhage [37]. The initial condition is often described as a burning or tingling sensation making the mouth hypersensitive to foods. And as the condition progresses, eating, swallowing, and talking become increasingly difficult [38]. In the more severe cases, OM can compromise the airway leading to anoxia-induced brain injury and even death [39–41]. As a form of iatrogenic stomatitis, mucositis usually starts off with aplasia 7–14 days after the initiation of chemotherapy. Clinically, the earliest sign may be characterized by leukoedema, appearing as a diffuse, poorly defined area of milky-white opalescence most noticeable on the buccal mucosa, which will disappear upon stretching [14]. In the following 1-2 weeks, a loss of epithelial structure and integrity is observed, and severe ulceration develops [42]. In general, OM can be assessed both clinically and with subjective input from the patient. The World Health Organization (WHO) has also provided a useful grading scale that combines both objective and subjective elements (Table 1) [43]. Current literature's reported incidence of OM is highly variable, ranging from 75% to 99% [44, 45]. The current working biophysical model of OM as proposed by Sonis [40] involves 5 phases: initiation, upregulation and message generation, amplification and signaling, ulceration, and healing. Initiation involves direct irreversible and reversible DNA damages and the generation of reactive oxygen species via the chemotherapeutic agents. In the upregulation and message generation phase, transcription factors (e.g., NF-*κ*B) are activated, resulting in the production of messaging and effector proteins including the proinflammatory cytokines and enzymes. Positive feedback loops in phase III increase cytokine production and thus signal amplification; apoptosis and tissue injury ensue. In the ulcerative stage, clinically evident erosions are detected; bacterial colonization and additional proinflammatory cytokine secretions are also involved. In due course, spontaneous healing occurs where epithelial cells migrate to cover the ulcerations [40]. Several chemotherapy agents have been associated with OM [46, 47]. A summary of these drugs is given in Table 2.

Chemotherapy-related oral infections, which account for 25–50% of the total infections, contribute significantly to the morbidity and mortality in these patients [48]. Susceptible areas include teeth, gingiva, salivary glands, and mucosa. It should be noted that in the myelosuppressed patient the cardinal signs of infection such as erythema and swelling are not always present. Therefore, the more reliable indicators such as fever, pain, and the appearance of lesions should be used to closely monitor all suspected infections [14]. Common oral flora and opportunistic microorganisms include coagulase-negative *Staphylococci* and *Streptococci*, *Klebsiella pneumonia, Pseudomonas aeruginosa, and Escherichia coli *[48–51]. It has been shown that pathogenic microorganisms found subgingivally or in periradicular area may cause acute exacerbations of preexisting periodontal or periradicular infections when the granulocyte count dips below 1000/mm^3^ [49, 52].

Perhaps, the most dangerous complication in the realm of infections comes from fungal species, most notably *Candida *species [48, 49]. The mortality rate from systemic fungal infections is much higher compared to other infections, with the majority believed to have originated from the oral cavity [49]. Clinically, fungal infections in the oral cavity can manifest in several forms, with erythematous or pseudomembranous candidiasis being the most common. Erythematous candidiasis presents itself as patchy or diffuse areas of erythema, often occurring on the palate. Pseudomembranous candidiasis appears as curd-like or patchy white lesions, which can be rubbed off but will produce bleeding and erosion in the tissue underneath. Also worth mentioning is hyperplastic candidiasis, which resembles leukoplakia as elevated white plaques that cannot be wiped off [53, 54]. Particularly troublesome is the chronic atrophic candidiasis, which is often accompanied by angular cheilitis and denture stomatitis. Angular cheilitis, an infection at the corner of the mouth, may sometimes involve *Staphylococcus* species. Ill-fitting denture bases, usually of the maxilla, can be a source of chronic irritation and a reservoir for *Candida albicans* [14].

Viral infections frequently seen in patients undergoing chemotherapy include the herpes simplex virus (HSV), varicella zoster virus (VZV), and cytomegalovirus (CMV) [14]. Viral reactivity is not uncommon during periods of myeloimmunosuppression, particularly with HSV infections. With incidence of recurrent infection reported up to 48%, HSV infected patients often report severe, painful, and prolonged ulcerations atypical of those discovered in immunocompetent hosts [55–57]. HSV recurrence typically appears 7–14 days after chemotherapy, and lesions can often be seen on lips and keratinized mucosa as small cluster of vesicles that rapidly ulcerate and coalesce [55, 58]. Fortunately, it is self-limiting and resolves in 2 weeks. VZV infections, also known as herpes zoster/shingles, can occur within the trigeminal dermatome. Lesions can be seen on the face or intraorally with the characteristic feature of halting abruptly at the midline on the side of the respective trigeminal divisions involved. Similar to HSV, VSV recurrence is confined to keratinized mucosa and is shown to manifest several weeks after the completion of chemotherapy, with widespread, painful lesions lasting up to several weeks. Intraorally, CMV infections may be seen as irregular pseudomembranous ulcerations, coupled with common clinical manifestations such as esophagitis, gastritis, colitis, hepatitis, pneumonia, and retinitis. Furthermore, a fever may also be involved but often resolves in 3–5 days. Dissemination of CMV in immunosuppressed patients is often fatal [14].

Intraoral bleeding is another complication associated with chemotherapy. The bleeding can be spontaneous, traumatically induced, or effect from existing pathology [59, 60]. It can also be the result of thrombocytopenia secondary to hematopoietic tissues suppression. Laboratory tests should be used to assess bleeding potential. Thrombocyte count and bleeding time can give the dentist a decent picture of the quantity, quality, and function of platelets.

### 3.4. Complications Associated with Radiotherapy

Orofacial tissues that may be influenced by head and neck radiotherapy include salivary glands, taste buds, mucous membranes, bone and teeth, the temporomandibular joint (TMJ), and related musculatures. In general, complications from radiation therapy are categorized into acute and chronic/late types. The acute effects usually develop early in the radiation treatment period and persist 2-3 weeks after completion of treatment, whereas the late effects may become evident at any time after treatment completion, ranging from weeks to years [61]. In fact, it is shown that in 90% to 100% of the patients whose radiation therapy covers the oral cavity some degree of oral complication will always develop [62].

Xerostomia is perhaps the most commonly reported oral sequela among patients receiving radiotherapy for head and neck cancers. The effects of radiation on salivary glands have been well documented. Ionizing radiation may cause irreversible damage to glandular tissue and loss of salivary fluid secretion; the gross architecture of the gland is slowly replaced by ductal remnants and fibrous tissues with lymphocytes and plasma cells infiltration [63, 64]. The progressive glandular atrophy and fibrosis and the reduction in salivary outflow occur shortly after the initial exposure to radiation and intensify thereafter [65]. Mantle, unilateral, and bilateral fields of radiation are associated with a reduction of salivary flow by 30% to 40%, 50% to 60%, and 80%, respectively. For patients whose major salivary glands are in the radiation fields, the prevalence of xerostomia is shown to range from 94% to 100% [66–68]. Clinically, the condition becomes apparent as saliva becomes “scant, sticky, and viscous.” The patient may experience oral discomfort and pain. Furthermore, dryness of the mucosa may put the patient at risk of oral infections and can lead to difficulties in speech, chewing, and swallowing, which significantly affects their quality of life [69, 70]. Reduced salivary outflow can also increase the susceptibility to dental caries and compromise the mucosal integrity [7]. It has been shown that xerostomia is associated with as little as two or three doses of 2 Gy each, whereas doses greater than 30 Gy can usually result in permanent or semipermanent xerostomia [71, 72]. It is interesting to note that a “compensatory” hypertrophy of the unirradiated salivary gland may occur after a few months and up to 1 year after therapy which may alleviate the condition; yet, if all the major salivary glands are included in the field of radiation, salivary function is expected to fall as much as 50–60% within the first week [73, 74]. Thus, it is suggested that, if irradiation of salivary tissues can be spared by patient positioning or shielding, the resultant salivary gland dysfunction may be reduced [75].

As taste is associated with salivary functions, it is not uncommon to hear complaints of taste loss in relation to xerostomia as a result of head and neck radiotherapy. Dysgeusia can occur at a rapid rate and be exacerbated at up to an accumulated dose of 30 Gy, then the progress of taste deterioration would slow down as perception for all four tastes, that is, salty, sweet, sour, and bitter, approaches zero [75]. In addition, microvilli damages brought about by the radiation may cause secondary taste loss [75]. Fortunately, the condition seems to be reversible. In the majority of the cases, taste acuity is reported to be partially restored and fully restored 20–60 days and 2–4 months after radiation therapy, respectively [76]. However, there were reports of subjective residual hypogeusia [75].

Perhaps, the most alarming and worrisome acute reaction for patients receiving radiotherapy is the radiation-induced mucositis. The high turnover rate and low radiation resistance of the mucosal cells within the oral cavity, pharynx, and larynx make them susceptible to destruction from head and neck fractionated external beam irradiation. In fact, the literature shows that mucosal erythema could develop within 1 week of fractionated doses of, each, 2 Gy per day [77]. The condition will intensify with continued treatment by daily regimen doses of greater than 2 Gy and large treatment volumes such that almost all patients would develop confluent mucositis by the third week [77]. Initially, the erythema is the result of epithelium thinning and vascular dilation, inflammation, and oedema of the submucosa [78]. As the therapy continues, however, the mucosa will become denuded, ulcerated, and covered with a fibrinous exudate [78]. There may also be bleeding. The patient is often accompanied by symptoms of intense pain, dysphagia, and odynophagia, which, in many cases, prevent oral intake and necessitate the use of parenteral analgesics; as a result, not only the patients' quality of life but also the implementation of the therapy itself is greatly affected [79]. Radiation-induced mucositis usually persists 2-3 weeks after completion of radiotherapy [77, 80]. About 90% to 95% of the patients would show complete resolution by the 4th week [77].

It is now widely accepted that, through the generation of free radicals, ionizing radiation can cause alteration of the vascular elements in the bone within the irradiated fields. Overtime, the irradiated area will show endothelial cell death, hyalinization, thrombosis, and obliteration of vessels; consequently, the periosteum and marrow spaces will become fibrotic while the osteoblasts and osteocytes will necrose [75]. The end result is an area described as being hypovascular, hypocellular, and hypoxic, with minimal ability to withstand trauma (e.g., dental extraction, alveoloplasty) or to be repaired [81–83]. However, it should be noted that the degree, progression, and irreversibility of these changes are thought to be dose related [75]. In fact, osteoradionecrosis (ORN) is not a common complication of radiotherapy, and the incidence in the literature has been reported to range from 1% to 37.5% [61]. A representative 30-year retrospective study of 830 patients showed a collective rate of only 8.2% [84]. There has also been report that the incidence of ORN is on a decline over the past 20 years, which may be explained by the advent of high-energy radiation sources [75, 85]. The ORN contains a wide range of clinical presentations which vary from a small stable asymptomatic region of exposed bone to a full-scale ORN that is accompanied by severe pain, foul-smelling necrotic bone of green-grey color, and suppuration [61]. In general, elective oral surgical procedures such as extractions or soft tissue surgeries are contraindicated within the irradiated field [7].

Currently, there is little data on the effects of ionizing radiation on teeth. Results from literature appear to be conflicting as to the differential decalcification rates between irradiated and nonirradiated teeth. However, it is widely agreed that the dental pulp of patients who received radiotherapy will demonstrate reduced vascularity, accompanied by fibrosis and atrophy [86]. Pulpal response to trauma, dental procedures, and bacterial assaults maybe compromised, but tolerance to pain seems to increase. The secretory mechanism of odontoblasts may also be affected as excessive osteodentine formation was observed in irradiated rats [87, 88]. With regard to tooth development, the timing of exposure is crucial; tooth bud may be destroyed if irradiation occurs before significant calcification, while growth may be retarded and enamel and dentine irregularities result if exposure happens during a later stage of development [86].

Under direct assault of the ionizing radiation, the TMJ and the muscles of mastication may ultimately undergo fibrosis and contracture resulting in trismus [89]. According to the literature, about 5% to 38% of the patients develop trismus after receiving radiation therapy for head and neck cancer [90, 91]. Clinically, trismus manifests as the gradual inability to open the mouth for normal functions; the onset of reduced interincisal opening is generally noted at 9 weeks after radiotherapy. A rate of 2.4% loss per month was observed in the following 9 months, and a 32% reduction in the mean interincisal opening was observed after 4 years [92]. Although speech articulation is not adversely affected, this painless condition could make mastication and oral intake of food particularly problematic. The implication is significantly more profound in the case of denture wearers as they may be unable to insert their prostheses and new ones cannot be satisfactorily made due to restricted access and range of jaw motions [75]. Oral hygiene is also severely compromised.

### 3.5. Complications Associated with HSCT

HSCT, once viewed as experimental decades ago, has since advanced as a customary treatment protocol for a variety of malignancies. HSCT can be categorized into allogenic and autologous: the former is where bone marrow is harvested from a histocompatible donor while the latter is from the patient's own; both are now regularly performed and considered standard of care for selected malignancies [93]. The risk of oral complications from HSCT is comparable to that of conventional chemotherapeutic treatment; patients receiving autologous transplant exhibit possibly slightly higher risk while those undergoing allogeneic graft may face cumbersome complications due to the infusion of donor's stem cells [94–96]. In general, patients undergoing HSCT are at high risk of bacterial (including those of periodontal origin), fungal (particularly *Candida*), and viral (e.g., HSV, VZV, and CMV) infections [97–106]. Also, there has been report of hairy leukoplakia in human-immunodeficiency-virus- (HIV-) negative HSCT patients [107]. Potentially life-threatening complications from preexisting periodontitis have also been implicated from cultures of atypical pathogenic organisms isolated from disease sites; the importance of establishing healthy periodontal status before cancer therapy could not have been emphasized more [108–110].

**Figure 4:**
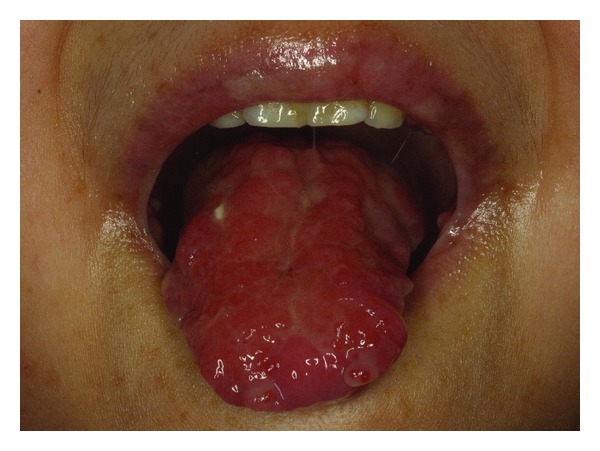
Soft tissue distortion in a patient with chronic GVHD.

Perhaps, of particular concern is the graft versus host disease (GVHD). GVHD is the most important complication of allogeneic transplantation. It occurs via an immunological reaction where the transplanted “graft” cells (donor lymphocytes) recognize the tissues of the “host” (the recipient) as foreign. GVHD can be acute or chronic [111]. Acute form of GVHD usually occurs within a few weeks of the transplantation. It damages the skin, gut, and liver. Typical signs and symptoms may include nausea, vomiting, abdominal pain, diarrhea, bloody stool, and jaundice [21]. The main risk factor is the major histocompatibility antigens (HLA) mismatch; it has been shown that, if prophylaxis is not provided, acute GVHD can affect almost every recipient [112]. Chronic GVHD may immediately follow the acute stage or may occur several months later. It is associated with loss of self-tolerance and symptomatically resembles scleroderma or Sjögren's syndrome [113]. Chronic GVHD is characterized by bronchiolitis, keratoconjunctivitis sicca, esophageal stricture, malabsorption, cholestasis, hematocytopenia, and generalized immunosuppression [21]. The oral manifestation of GVHD varies with the severity of the condition and is associated with a spectrum of presentations. In general, clinical or subclinical chronic GVHD exhibits features that include mild oral mucosal erythema, desquamative gingivitis, loss of lingual papillae, lichenoid hyperkeratosis, and xerostomia (Figure 4), whereas acute GVHD patients may encounter painful desquamation and ulcerative-pseudomembranous reactions. Erythema, angular cheilitis, and lichenoid-like changes have also been observed [111].

## 4. Present Practice and Therapeutic Options 

Although priority is often given to the treatment of the malignancy itself, focus should also be directed at prevention and amelioration of complications that may occur as a result of the disease and/or its treatment. A thorough head and neck evaluation, oral soft and hard tissue examination, and the associated intraoral radiographs are all essential parts of the initial dental visit for cancer patients. The goal of such visit is to remove and document any preexisting acute and chronic pathological conditions, for example, periodontal and periapical pathology, residual cysts, and impacted or partially erupted teeth. Through consultation with the patient's primary-care physician and revision of his/her medical status, oral surgery, intermediate or definitive restorations, and oral prophylaxis procedures may be performed safely, and if required, under intravenous sedation and/or local/general anesthesia [7]. It should be noted that evaluation, treatment, and prevention of any preexisting oral and dental pathology contribute significantly to the overall favourable treatment outcome for cancer patients; for this reason, the patient's oral health status should be stabilized/optimized for minimally predictable complications [7, 114].

In the following section, some therapeutic options for the management of common oral complications of cancer treatment are presented. Through this general guide, the author of this paper sincerely hopes that general dental practitioners can benefit from it and may find it useful in ameliorating some of the painful oral complications of cancer therapy.

### 4.1. Mucositis

Presently, there is no medication proven to be able to successfully eliminate mucositis [4]. However, painful symptoms can still be managed and oral discomfort alleviated so as to improve the patient's quality of life. The current approach focuses on the management of pain and the encouragement of eating, especially in chemotherapy-induced mucositis [115]. One strategy on pain relief pertains to the use of an oral solution mixture known as “Magic Mouthwash;” it is composed of diphenhydramine, viscous lidocaine, bismuth, subsalicylate, and corticosteroids [116]. It is said to relieve acute pain and reduce inflammation, making oral consumption of food much easier. Yet, high-grade mucositis pain is commonly relieved with potent analgesic medications such as opioids [117]. Alternatively, a number of recent studies have investigated the potential of newer therapeutic interventions, in particular, concerning the efficacy of growth factors and cytokines in curtailing the development of high-grade mucositis and reducing the duration of the lesions. Palifermin (Kepivance; Amgen, Thousand Oaks, CA, USA), a recombinant human keratinocyte growth factor vigorously researched, shows much promise in reducing the frequency of high-grade (WHO grade 3 or 4) mucositis [118]. Furthermore, palifermin has been demonstrated to decrease the duration of mucositis, thereby lessening the use of parenteral nutrition and entailing higher scores for physical and functional well-being [118]. In a separate study, the beneficial effect of palifermin as preventive therapy for mucositis has been confirmed [119]. Nevertheless, the drug is not without its side effect; taste alteration in patients treated with palifermin has been reported [120].

Other practical therapies for mucositis are also showing promises, though evidence and data from the literature are limited. One method widely used among oncologists is the application of ice chips to the mouth every 30 minutes for prevention and treatment of oral mucositis in patients undergoing chemotherapy. The rationale of oral cryotherapy is that, through vasoconstriction, the release of chemotherapeutic drugs to the mucosal epithelium is reduced [121]. Perhaps, an effective preventive measure of recent interest is the use of low level laser therapy (LLLT). Various studies have demonstrated the potential benefits of LLLT in its ability to reduce the rates of WHO severe grade mucositis [121, 122]. Other novel methods and experimental approaches include a formulation containing the amino acid L-glutamine and the hormone, leptin; both have been shown to have a positive impact on the development of mucositis [123–126].

### 4.2. Oral Infections

Often, cancer treatments can negatively affect the patient's immune system. With his/her immune response suppressed, opportunistic infections can contribute to significant morbidity and mortality.

#### 4.2.1. Bacterial Infections

Normal oral flora comprises of a variety of bacteria, some of which may become pathogenic with immunosuppression. According to Rautemaa and colleagues, sepsis of unknown origin may possibly be the result of oral infections (e.g., Viridans* Streptococcus*, *Prevotella* species, *Fusobacterium*, *Actinobacillus actinomycetemcomitans*, and *Actinomyces* species) [127]. Nonetheless, the infections are usually localized to oral mucosa and can be treated with a combination of penicillin and metronidazole, followed by routine dental procedures if necessary [4]. Given the patient's condition, meticulous oral hygiene practice is paramount. Bacteria may be removed from the teeth by gentle brushing with a soft bristle tooth brush and flossing. One may consider using an antimicrobial mouthwash as an adjunct. In the case where brushing becomes difficult (e.g., mucosal damage), a chlorhexidine-containing mouthwash is generally recommended [6].

#### 4.2.2. Candidiasis

According to Lalla and colleagues, the prevalence of oral fungal infection from all forms of cancer therapy was about 7.5% before treatment, 40% during treatment, and 30% after treatment [128]. Yet, a Cochrane meta-analysis has concluded that there is currently insufficient evidence from the literature to make a recommendation for or against the treatment of oral candidiasis with antifungal agents in patients undergoing cancer treatment [129]. Nevertheless, it is the responsibility of the general dental practitioner to ease the patient's suffering, and that entails morbidity reduction and systemic infection prevention. It should be noted that although topical antifungal agents are commonly prescribed for their lower risk of side effects and drug interactions, literature support of their efficacy is inconsistent [128]. According to the guidelines provided by the Infectious Disease Society of America (IDSA), clotrimazole troches and nystatin pastilles are the first line drugs for mild oropharyngeal candidiasis [130]. However, they may be difficult to apply in situations such as hyposalivation and/or mucositis in which the experience can be traumatic; thus, an alternate solution is to use nystatin rinses [128]. With the high relapse rate of topical agents, one may also consider systemic antifungal agents [131]. In fact, the IDSA guidelines recommend the use of systemic fluconazole (100–200 mg/day for 2 weeks) (Diflucan; Pfizer Labs, New York, NY, USA) for the management of moderate to severe infections [130]. In fluconazole resistant cases, itraconazole capsules (200 mg/day for 2–4 weeks) or itraconazole oral solution (200 mg/day for 2 weeks) may also be considered [128]. As a second line drug, posaconazole (Noxafil; Merck & CO., Whitehouse Station, NJ, USA) is suggested by the IDSA [130]. In situations where the disease becomes refractory, a broader spectrum drug of a more potent nature such as voriconazole (Vfend; Pfizer Labs, New York, NY, USA), caspofungin (Cancidas; Merck & CO., Whitehouse Station, NJ, USA), and amphotericin B (Fungizone; Bristol-Myers Squibb Co., Princeton, NJ, USA) is suggested. Note that voriconazole has been reported to be associated with severe photosensitivity, and possibly an increased risk of skin cancer, whereas amphotericin B is known for its systemic side effects, for example, high fever [128, 130, 132].

Whereas the aforementioned modalities are all aimed at treating oral candidiasis, the potential benefits of prophylaxis should not be ignored, particularly in severely immunosuppressed and/or neutropenic patients. From a Cochrane review, there is reasonably good evidence from randomized controlled trials that drugs absorbed from the GI tract prevent candidiasis in cancer patients [129]. A number of studies have demonstrated the efficacy of prophylactic use of fluconazole, itraconazole, posaconazole, and intravenous micafungin (Mycamine; Astellas Pharma US, Inc., Deerfield, IL, USA) in reducing the prevalence of all clinical fungal infections during cancer therapy [128, 133–137]. Interestingly, however, there has been little evidence from the literature on the relative cost effectiveness of systemic versus topical prophylaxis for oral fungal infections [129].

#### 4.2.3. Viral Infections

HSV is quite prevalent in the general population. In the majority of the cases, HSV infection stems from latent viral reactivation. Current literature suggests that immunosuppression due to chemotherapy is the main contributive factor, with prevalence approaching 40%. Neutropenic patients with hematological malignancies are at the greatest risk, that is, 50%, during treatment [138]. Presently, acyclovir (Zovirax; GlaxoSmithKline Pharmaceuticals, Research Triangle Park, NC, USA) and valacyclovir (Valtrex; GlaxoSmithKline Pharmaceuticals, Research Triangle Park, NC, USA) have both been shown to be equally efficacious in prevention and treatment of HSV [139]. Oral prophylaxis can be accomplished with acyclovir at the dose of 200–800 mg thrice a day or valacyclovir at the dose of 500 mg twice a day [139–141]. During treatment, acyclovir may be used intravenously at the dose of 5 mg/kg every 8 hours or perorally 200–400 mg 3–5 times a day; on the other hand, the unavailability of intravenous valacyclovir limits its use to the oral dosing regimen of 500–1000 twice a day [139]. An alternative is famciclovir (Famvir; Novartis Pharmaceuticals Corp., East Hanover, NJ, USA). In case of drug resistance, intravenous foscarnet (Foscavir; AstraZeneca, Wilmington, NC, USA) and cidofovir (Vistide; Gilead Sciences, Inc., Foster City, CA, USA) may be used [142].

Of particular concern is oral hairy leukoplakia (OHL), a result of Epstein-Barr virus infection, commonly seen in HIV infected individuals. However, OHL can also manifest in immunocompromised patients (e.g., patients under cancer treatment) who are HIV negative. Reports have shown that OHL can occur in patients under chemotherapy for acute myelogenous leukemia, acute lymphocytic leukemia, and multiple myeloma, as well as in patients under corticosteroid regimen for gastrointestinal stromal tumor [143–147]. At present, there is no universal therapy for the management of OHL; however, high dose oral valacyclovir may be used safely and effectively [148]. Alternatively, topical treatment, that is, 25% podophyllin resin alone or in combination with 5% topical acyclovir, and gentian violet could be considered [149–151].

### 4.3. Xerostomia

Patients who have undergone head and neck cancer radiotherapy tend to have long-term side effects which are xerostomia and hyposalivation, resulting in further complications such as increased caries incidence and loss of taste [5]. It is advisable for the patient who has dry mouth to take frequent sips of water (every 10 minutes) and melt ice chips in mouth for comfort. Additionally, one may consider the use of artificial saliva spray (e.g., Xerotin, Moi-Stir, Salivart, Xero-Lube, Saliva Orthana) and mouth moisturizing gel (e.g., Biotène Oral Balance). The lips may well be lubricated with petroleum jelly or a lanolin-containing preparation (e.g., BioXtra moisturizing gel). Patients should be cautioned against coffee, tea, soft drinks with caffeine, and commercial mouth rinses with alcohol as they can dehydrate the mouth. Alcohol-free mouth rinses (e.g., BioXtra alcohol-free mouthrinse, Biotène mouthwash, and OralSeven moisturising mouthwash) are recommended. Residual salivary gland activity and salivary flow rate may be increased by saliva stimulating tablets (SST) and medications like pilocarpine (Salagen, 5 mg, thrice a day), respectively. Finally, patients are recommended to use sorbitol- or xylitol-based chewing gum for salivary flow stimulation and caries arresting.

### 4.4. Dysgeusia

It is estimated that about 50% to 75% of the cancer patients receiving chemotherapy, radiotherapy, or both will suffer from distorted or impaired ability to taste [152, 153]. While patients under radiation treatment tend to suffer most from dysgeusia, its severity is highly correlated to the cumulative radiation dose. Mild dysgeusia is generally well tolerated; nevertheless, impaired ability to taste, which affects appetite, reduces caloric intake, induces weight loss, and hampers nutritional status, can exert great impact on the patient's quality of life [5]. At present, several strategies have been proposed for the management of dysgeusia. Although clinical efficacy of zinc supplementation has been quite variable, its use has been suggested by several studies to ameliorate the debilitating effects of dysgeusia [154]. The rationale is such that zinc element may be structurally important in the proteins responsible for regulating the taste bud pores [5]. Additionally, one may also consider supplementing diet with vitamin D as it was reported that patients suffering from dysgeusia made improvement from it [155]. Sometimes, dietary counselling may have more impact on long-term dysgeusia and improve patient outcome [156, 157]. Several simple methods have been utilized by nutritionists for symptomatic patients [153]. Patients are advised to drink plenty of fluids during meal, as such would enable the dissolution of taste components in the food and facilitate their translocation to taste buds. Food should be chewed slowly and thoroughly to release more flavours and stimulate saliva production; this is especially crucial if the patient is also suffering from dry mouth where saliva is important to taste. Patients should also switch foods during meals to prevent adaptation of taste receptors while taking care to maintain a balanced diet at the same time [152].

## Figures and Tables

**Table 1:** WHO oral mucositis scale [43].

Grade	Clinical presentation
0	Normal
1	Soreness with/without erythema
2	Ulceration and erythema
3	Ulceration and extensive erythema, patient cannot swallow solid food
4	Mucositis of such severity that feeding is not possible

**Table 2:** A summary of mucosatoxic chemotherapy agents (data adapted from Köstler et al., 2001 [46], and Saadeh, 2005 [47]).

Category	Drugs
Alkylating agents	Busulfan, carmustine, chlorambucil, cisplatin, cyclophosphamide, dacarbazine, estramustine, ifosfamide, lomustine, mechlorethamine, melphalan, oxaliplatin, procarbazine, and thiotepa
Anthracyclines	Daunorubicin, doxorubicin, epirubicin, idarubicin, and mitoxantrone
Antimetabolites	Capecitabine, cytarabine, floxuridine, 5-fluorouracil, hydroxyurea, 6-mercaptopurine, methotrexate, pemetrexed, and 6-thioguanine
Antitumor antibiotics	Actinomycin d, amsacrine, bleomycin, mithramycin, mitomycin, and plicamycin
Natural products	Etoposide, irinotecan, and streptozotocin
Taxanes	Docetaxel and paclitaxel
Vinca alkaloids	Vinblastine, vincristine, vindesine, and vinorelbine
Others	Carboplatin, fludarabine, gemcitabine, interferons, interleukin-2, mitotane, and topotecan
